# Multi-omics analysis reveals gut microbiota-host transcriptomic remodeling associated with the protective effects of Lacticaseibacillus rhamnosus GG against rotavirus enteritis

**DOI:** 10.3389/fcimb.2026.1901881

**Published:** 2026-08-05

**Authors:** Xiaoling Lin, Runjin Zhou, Bing Zhu, Haiyang He, Xiaogang Chen

**Affiliations:** 1 Guangzhou Women and Children’s Medical Center, Guangzhou Medical University, Guangzhou, China; 2 The First Clinical Medical College of Guangzhou University of Chinese Medicine, Guangzhou, China; 3 Dongguan Hospital of Traditional Chinese Medicine, Dongguan, China; 4 Department of Immunology, Basic Medical School, Army Medical University (Third Military Medical University), Chongqin, China; 5 The First Affiliated Hospital, Guangzhou University of Traditional Chinese Medicine, Guangzhou, China

**Keywords:** rotavirus enteritis, gut microbiota, *Lacticaseibacillus rhamnosus* GG, mucosal immunity, multi-omics, transcriptomics

## Abstract

**Objective:**

Rotavirus enteritis is closely associated with intestinal epithelial injury, gut microbial dysbiosis, and mucosal immune perturbation; however, the microbiota-host mechanisms underlying the protective effects of Lacticaseibacillus rhamnosus GG (LGG) remain incompletely defined. This study aimed to characterize LGG-induced microbiota-host transcriptomic remodeling in neonatal mice with rotavirus enteritis using an integrated multi-omics framework.

**Methods:**

BALB/c suckling mice were assigned to Control, Model, and LGG groups. Rotavirus enteritis was induced by oral gavage with SA11 rotavirus, and mice in the LGG group received oral LGG intervention for 7 consecutive days. Diarrhea symptoms and jejunal histopathological changes were evaluated. Gut microbial composition was analyzed using 16S rRNA sequencing, while jejunoileal transcriptional responses were profiled by RNA sequencing. Differentially abundant bacterial taxa, differentially expressed genes, KEGG-enriched pathways, mucosal B-cell/IgA-related transcriptional modules, and integrated Genus-Gene-Pathway networks were further analyzed.

**Results:**

LGG alleviated rotavirus-induced diarrhea, dehydration, body weight loss, and jejunal mucosal injury. Rotavirus infection disrupted the gut microbial ecosystem, with reduced α-diversity, altered community structure, increased *Rodentibacter* and *Proteus*, and decreased *Muribacter*, *Alistipes*, *Paraclostridium*, and *Tuzzerella*; these dysbiotic changes were partially reversed by LGG. Transcriptomic analysis identified 323, 91, and 552 differentially expressed transcripts in the Control vs Model, Model vs LGG, and LGG vs Control comparisons, respectively. Rotavirus infection mainly activated infection-related, inflammatory, endoplasmic reticulum stress, NF-κB, and MAPK signaling pathways, whereas LGG intervention was associated with epithelial repair, cytoskeletal remodeling, cell-death clearance, nucleocytoplasmic transport, and Notch signaling. Rotavirus infection also enhanced mucosal B-cell/IgA-related transcriptional programs, while LGG reduced *Ighv3–8* and *Ighv4–2* expression and tended to attenuate B-cell class-switching, plasma-cell differentiation, and IgA-production module scores. Integrated Genus-Gene-Pathway analysis identified 645 significant genus-gene correlations, revealing that LGG-associated microbial alterations were associated with host pathways related to epithelial barrier repair, immune regulation, PI3K-Akt/Rap1 signaling, iron metabolism, and ferroptosis.

**Conclusion:**

LGG may promote the restoration of intestinal mucosal homeostasis after rotavirus infection by modulating coordinated changes between specific gut bacterial genera and host transcriptional programs related to mucosal repair and immune homeostasis. These findings provide integrated multi-omics evidence for microbiota-host interaction mechanisms underlying LGG-mediated protection against rotavirus enteritis.

## 1 Introduction

Rotavirus infection remains a major cause of acute gastroenteritis and severe diarrhea in children under 5 years of age worldwide. Although rotavirus vaccination has markedly reduced severe disease, hospitalization, and mortality, rotavirus-associated diarrhea continues to impose a substantial burden (Aliabadi et al., 2019), particularly in low- and middle-income countries. This persistent burden is influenced by regional differences in vaccine coverage, nutritional status, sanitation, and gut microbial ecology, which may affect both disease severity and vaccine-derived protection (Troeger et al., 2018). Thus, rotavirus infection remains an important challenge in pediatric infectious disease prevention and control.

No specific antiviral therapy is currently available for rotavirus enteritis, and management remains largely supportive. Probiotics, particularly Lacticaseibacillus rhamnosus GG (LGG), have been explored as adjunctive treatments for pediatric acute gastroenteritis. However, current guidelines provide only weak recommendations with low-certainty evidence, and systematic reviews and large trials have reported heterogeneous efficacy (Szajewska et al., 2023). Although experimental studies suggest that LGG may alleviate rotavirus-induced epithelial injury (Schnadower et al., 2018), its *in vivo* regulation of the gut microbiota, epithelial responses, and mucosal immune networks remains insufficiently understood.

Rotavirus infection induces diarrhea not only by damaging mature absorptive epithelial cells in the small intestinal villi and impairing water-electrolyte absorption, but also by disrupting gut microbial homeostasis. Clinical studies have shown that infected children exhibit reduced microbial diversity, distinct microbial community structures, and increased abundance of potentially pathogenic bacteria (Sohail et al., 2021), while animal studies further suggest that rotavirus alters mucosal glycan availability and small intestinal ecological niches, leading to site-specific changes in the ileal microbiota (Engevik et al., 2020). Conversely, the pre-existing gut microbiota can modulate host susceptibility to rotavirus infection, antiviral immunity, and oral vaccine immunogenicity, indicating a bidirectional interaction among rotavirus, the gut microbiota, and mucosal immunity (Twitchell et al., 2016; Kim et al., 2020). As the jejunum and ileum are central sites for absorption, barrier maintenance, antiviral defense, and mucosal adaptive immunity, transcriptomic profiling of jejunoileal tissues provides an opportunity to systematically characterize local host responses, including epithelial repair, interferon-mediated antiviral signaling, inflammatory regulation, barrier remodeling, B-cell/IgA-related immunity, and metabolic reprogramming. These responses are likely organized in a cell type-specific and network-dependent manner, as suggested by recent single-cell transcriptomic studies (Sen et al., 2012; Bomidi et al., 2021; Triana et al., 2021).

Previous studies on LGG intervention in rotavirus infection have largely relied on single-omics approaches. Although 16S rRNA sequencing can characterize microbial dysbiosis and ecological remodeling, it cannot directly explain host mechanisms related to epithelial repair, barrier maintenance, and mucosal immune regulation. Conversely, jejunoileal transcriptomics can reveal host gene-expression changes and signaling pathways, but cannot determine whether these responses are linked to specific microbial alterations without matched microbiota data. Therefore, integrative microbiome-transcriptome analysis offers a more comprehensive strategy for elucidating host-microbiota interactions under probiotic intervention (Wang et al., 2019). In this study, we evaluated the effects of LGG in a suckling mouse model of rotavirus enteritis by combining 16S rRNA sequencing with jejunoileal transcriptomic profiling. This approach aimed to identify key bacterial genera, host genes, and signaling pathways associated with antiviral responses, mucosal barrier repair, inflammatory regulation, and immune modulation, thereby clarifying the microbiota-host mechanisms through which LGG alleviates rotavirus enteritis.

## 2 Materials and methods

### 2.1 Animal model

Three- to four-day-old BALB/c suckling mice were randomly assigned to the control group, model group, and Lacticaseibacillus rhamnosus GG (formerly Lactobacillus rhamnosus GG; LGG) group, with six mice in each group. The pups were nursed by their dams. Model induction was performed when the pups reached 7 days of age. On experimental days 1-3, mice in the model group and LGG group were orally gavaged with 100 μL of SA11 rotavirus suspension per mouse at a concentration of 1 × 10^7 FFU/mL once daily for 3 consecutive days. Mice in the control group received an equal volume of PBS by oral gavage. On experimental days 1-7, mice in the control and model groups were orally administered 100 μL of PBS per mouse once daily, whereas mice in the LGG group were orally administered 100 μL of LGG suspension per mouse at a concentration of 7.7 × 10^9 CFU/mL once daily for 7 consecutive days.

### 2.2 Virus preparation

Rotavirus SA11 was kindly provided by Professor Haiyang He from the Department of Immunology, Army Medical University of the Chinese People’s Liberation Army. The SA11 virus was activated with trypsin at 10 μg/mL at 37 °C for 40 min and then used to infect confluent MA104 cell monolayers for 1 h. The inoculum was subsequently replaced with serum-free medium, and the cells were cultured for approximately 48 h until 90% cytopathic effect was observed. The cultures were subjected to 2–3 cycles of freezing and thawing. PEG 6000 was then added to a final concentration of 10%, followed by stirring at 4 °C for 6–8 h. The suspension was centrifuged at 20,000 × g for 30 min at 4 °C, and the resulting pellet was resuspended in PBS at 1/20 of the original volume, aliquoted, and stored at -80 °C.

### 2.3 Virus titration

The virus suspension was vortexed thoroughly and serially diluted from 10^-2 to 10^-6. The diluted virus samples were activated with 10 μg/mL trypsin without EDTA at 37 °C for 40 min and then used to infect fully confluent MA104 cell monolayers for 1 h. The virus inoculum was removed, and the cells were cultured in serum-free DMEM for 18-20 h. The cells were subsequently fixed with 4% paraformaldehyde, permeabilized with 1% Triton X-100, treated with 100 mmol/L ammonium chloride, and washed. FITC-conjugated anti-rotavirus antibody diluted at 1:100 was added, followed by incubation in the dark for 2 h. After washing, fluorescent foci were detected using an immunofluorescence spot analyzer. The viral titer was calculated according to the focus-forming assay (FFA) formula: Viral titer (FFU/mL) = mean number of fluorescent cells per well at the appropriate dilution × dilution factor/inoculation volume. The appropriate dilution was defined as the dilution at which the number of fluorescent cells per well ranged from 10 to 100.

### 2.4 Histopathological examination

On experimental day 8, suckling mice were euthanized by overdose inhalation of veterinary isoflurane. In brief, 1 mL of isoflurane was applied to a cotton ball placed in a sealed induction chamber. After 1 min of equilibration, the mice were introduced into the chamber and exposed to isoflurane vapor until loss of consciousness, followed by progressive respiratory and cardiac depression and complete cessation of respiration and heartbeat. Jejunal segments were collected for histopathological analysis. Jejunal tissues were fixed in 4% paraformaldehyde for more than 24 h, followed by dehydration through a graded ethanol series, clearing in xylene, paraffin infiltration, and embedding. Sections of 3-5 μm thickness were prepared, mounted on slides, and dried at 60 °C. After deparaffinization and rehydration, the sections were stained with hematoxylin for nuclear visualization, differentiated with hydrochloric acid alcohol, blued, and counterstained with eosin for cytoplasmic staining. The sections were then dehydrated through graded ethanol, cleared in xylene, mounted with neutral resin, and examined microscopically after solidification.

Histological injury was assessed using a semi-quantitative scoring system ranging from 0 to 4. A score of 0 indicated normal intestinal architecture; 1 indicated mild mucosal and submucosal edema; 2 indicated moderate mucosal and submucosal edema; 3 indicated severe mucosal and submucosal edema accompanied by focal villus shedding; and 4 indicated severe mucosal and submucosal edema with villus necrosis and detachment, as well as complete disruption of intestinal architecture.

### 2.5 16S rRNA gene sequencing

Suckling mice were anesthetized with isoflurane, and colorectal contents were collected using sterile forceps. The samples were mixed with grinding beads and 1 mL of Buffer ATL/PVP-10, homogenized, and lysed at 65 °C for 20 min. After centrifugation, the supernatant was collected, mixed with 600 μL of Buffer PCI, and centrifuged again. The resulting supernatant was transferred to a deep-well plate preloaded with magnetic bead-binding solution, proteinase K, and RNase A. DNA extraction and washing were performed using a KingFisher automated extraction system, and DNA was eluted with Elution Buffer. The extracted DNA was used for bacterial 16S rDNA meta-amplicon sequencing. The workflow included sample quality assessment, PCR amplification, library quality control, and high-throughput sequencing.

### 2.6 Intestinal transcriptome sequencing

Intestinal tissues were lysed with TRIzol reagent, followed by chloroform extraction, isopropanol precipitation, and washing with 75% ethanol. After air-drying, the RNA pellet was dissolved in DEPC-treated water. Subsequently, mRNA was enriched using oligo(dT) beads, fragmented, and reverse-transcribed to synthesize cDNA. After adaptor ligation and library enrichment, the constructed libraries were sequenced on an Illumina NovaSeq platform.

### 2.7 16S rRNA data processing and differential abundance analysis

Raw paired-end 16S rRNA sequencing reads were processed using the DADA2 pipeline for quality control, denoising, sequence merging, and chimera removal. Taxonomic annotation was performed against the SILVA v138.1 database. The resulting amplicon sequence variant (ASV) count table was subsequently aggregated to generate genus- and family-level abundance matrices. Alpha diversity was assessed using the Shannon index and observed ASVs. Beta diversity was evaluated based on Bray-Curtis distances, visualized by principal coordinates analysis (PCoA), and tested using permutational multivariate analysis of variance (PERMANOVA) to determine differences in microbial community structure among the three groups. Differential abundance analysis at the genus level was performed using DESeq2. Statistical significance was uniformly defined as an adjusted *P* value (*padj*) < 0.05 and an absolute log2 fold change ≥ 1.

### 2.8 RNA-seq differential analysis and KEGG pathway enrichment

Jejunoileal RNA-seq data were first quantified using Salmon, and differential expression results for Model vs Control, LGG vs Model, and LGG vs Control comparisons were subsequently obtained using DESeq2. Strictly differentially expressed transcripts were defined as those with an adjusted *P* value (*padj*) < 0.05 and an absolute log2 fold change ≥ 1. The differentially expressed transcripts were further subjected to KEGG pathway enrichment analysis using clusterProfiler to identify functional pathway modules associated with the disease state and LGG intervention.

### 2.9 Mucosal B cell/IgA-related transcriptional module scoring

Based on the DESeq2-normalized expression matrix, transcripts were aggregated at the gene-symbol level. Sample-level module scores were then calculated for three predefined gene sets related to B-cell class-switch recombination, plasma cell differentiation, and IgA production. Module scores were generated by applying within-gene z-score normalization followed by averaging across genes within each module. Intergroup differences were assessed using the Kruskal-Wallis test and pairwise Wilcoxon tests. In addition, sample-level heatmaps were generated for key genes to evaluate whether LGG-associated mucosal humoral immune features formed a stable expression pattern.

### 2.10 ASV-gene correlation analysis and genus-gene-pathway integration

In this study, 16S rRNA sequencing and RNA-seq data were aligned at the sample level. Spearman correlation analysis was used to construct an ASV-gene association matrix, which was further summarized at the Genus-Gene level by integrating taxonomic annotations. Strongly correlated pairs were retained using thresholds of |rho| ≥ 0.7 and correlation FDR < 0.05. These pairs were then integrated with RNA-seq differentially expressed genes and KEGG pathways to establish candidate Genus-Gene-Pathway axes. This integrative framework was used to characterize the coupling patterns between key LGG-associated microbial nodes and host functional pathways.

### 2.11 Statistical analysis and visualization

Normally distributed data were analyzed using one-way analysis of variance, followed by pairwise *post hoc* comparisons. Non-normally distributed data were analyzed using the Kruskal–Wallis test, followed by pairwise Wilcoxon rank-sum tests. All multiple comparisons were corrected using the Benjamini–Hochberg method, and an adjusted P value < 0.05 was considered statistically significant. Data visualization was primarily performed using R and Python.

## 3 Results

### 3.1 LGG alleviates rotavirus enteritis

**Figure 1:**
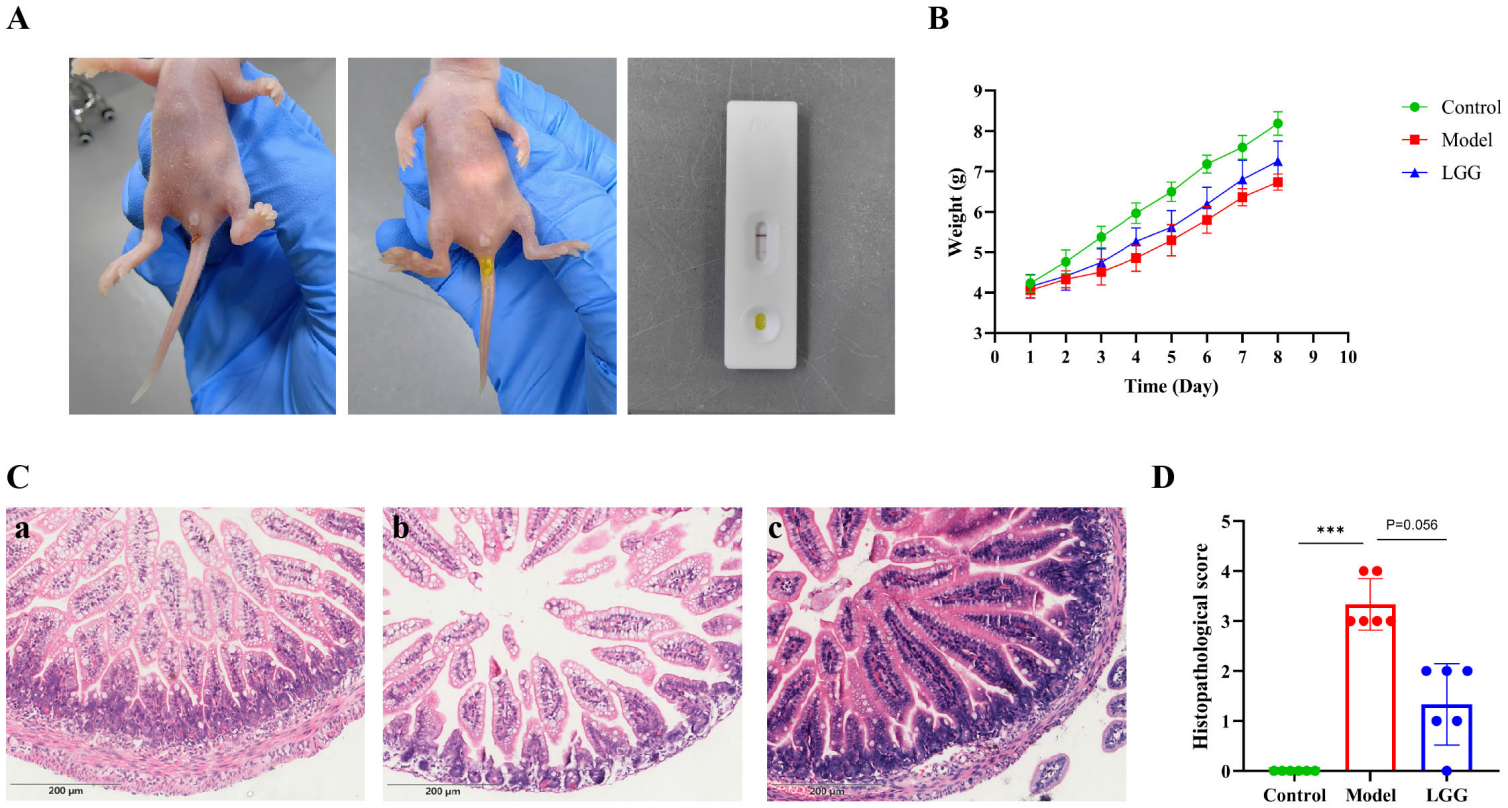
LGG alleviated rotavirus enteritis. **(A)** Seven-day-old BALB/c neonatal mice developed diarrhea after infection with the SA11 rotavirus strain, and the results of the colloidal gold immunochromatographic assay were positive. **(B)** Body weight gain of neonatal mice in each group. **(C)** Hematoxylin and eosin (H&E) staining of jejunal tissues from neonatal mice. a, Control group; b, Model group; c, LGG group. **(D)** Histopathological scores of intestinal tissues in each group. The symbol *** means "P < 0.001."

After oral administration of rotavirus, suckling mice developed egg-drop-like and watery stools, accompanied by abdominal distension, wrinkling of the abdominal skin, body weight loss, and perianal erythema and swelling (**Figure 1A**). Some pups also exhibited a decrease in body temperature. Hematoxylin and eosin staining showed marked edema and degeneration of the intestinal villous epithelial cells. Extensive villous necrosis and shedding were observed, with disorganized and irregular villous architecture. Pronounced vascular dilation and congestion were present in the mucosal and submucosal layers, accompanied by scattered mild lymphocytic infiltration.

After 1 week of LGG intervention, the diarrhea symptoms were alleviated compared with those in the model group, and dehydration was also less severe. Body weight increased to some extent (**Figure 1B**). HE staining of the jejunum showed only mild edema and degeneration of the villous epithelial cells, slightly disordered villous arrangement, and mild vascular dilation and congestion in the mucosal and submucosal layers, accompanied by relatively abundant lymphocytic infiltration (**Figure 1C**). These findings suggest that LGG effectively alleviated diarrhea symptoms and intestinal injury induced by rotavirus infection. Histopathological scoring further showed that intestinal injury was reduced after LGG intervention compared with the model group (**Figure 1D**).

### 3.2 LGG reshapes the gut microbial community structure

**Figure 2:**
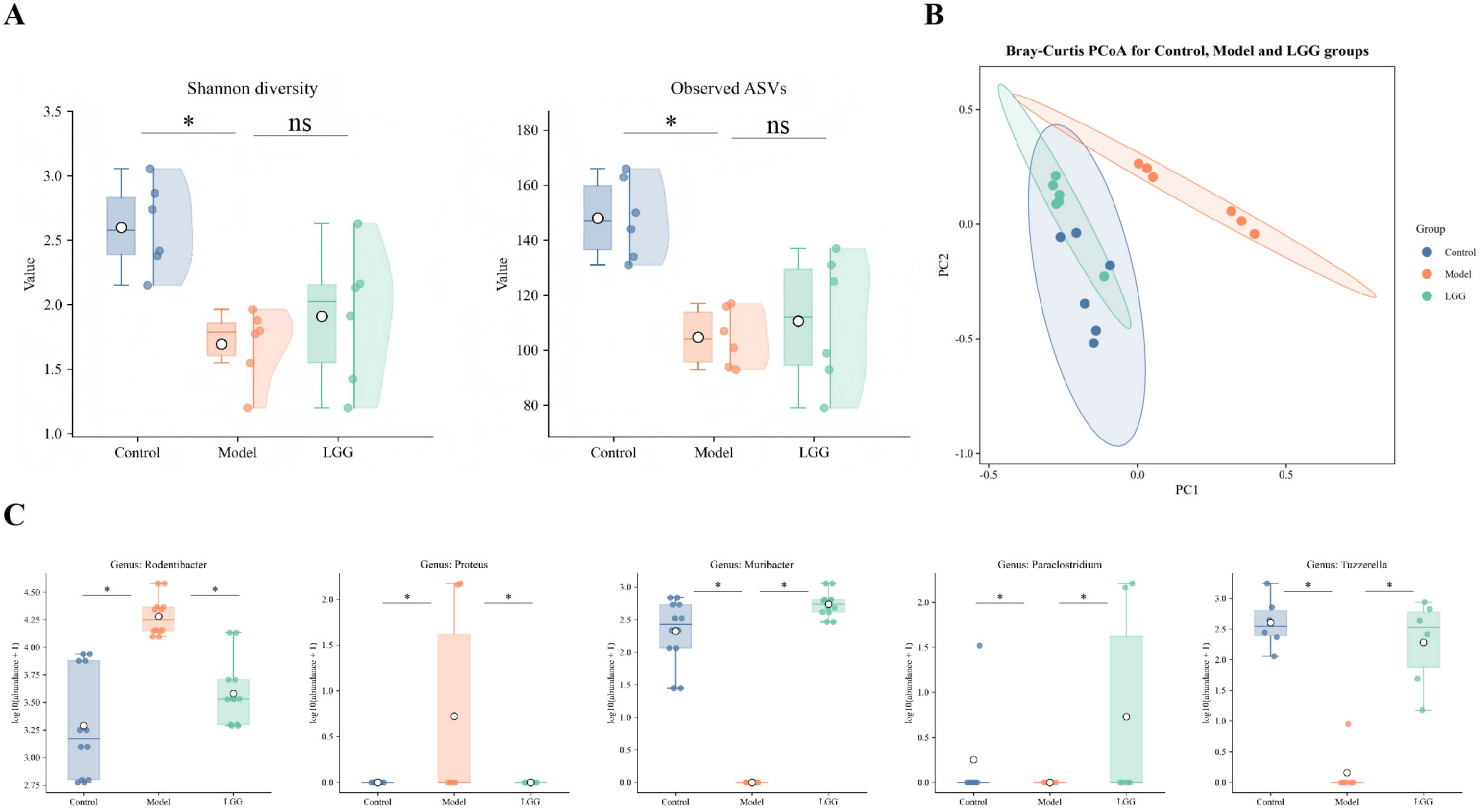
LGG Reshapes the gut microbial community structure. **(A)** Gut microbiota α-diversity. **(B)** PCoA based on Bray-Curtis dissimilarity. **(C)** Differential bacterial taxa. The symbol * means "P < 0.05", and ns indicates no significant difference.

In the alpha diversity analysis, the mean Shannon index values in the Control, Model, and LGG groups were 2.60, 1.69, and 1.91, respectively, while the mean numbers of observed ASVs were 148.0, 104.7, and 110.7, respectively (**Figure 2A**). Compared with the Control group, the Model group showed significant reductions in both the Shannon index and observed ASVs (*P* = 0.006). Compared with the Model group, the LGG group exhibited modest increases in both the Shannon index and observed ASVs; however, these differences were not statistically significant (*P* = 0.310 and *P* = 0.748, respectively). Principal coordinate analysis (PCoA) based on Bray-Curtis distances showed clear separation between the Control and Model groups in the principal coordinate space, whereas the LGG samples tended to shift away from the Model group and toward the Control group (**Figure 2B**). PERMANOVA further indicated that group assignment explained 44.39% of the variation in microbial community structure (*R²* = 0.444, *F* = 5.987, *P* = 0.001).

A total of 19 differentially abundant genera were identified between the Control and Model groups. Compared with the Control group, the Model group showed significant increases in *Rodentibacter* and *Proteus*, whereas *Muribacter*, *Alistipes*, *Paraclostridium*, and *Tuzzerella* were significantly decreased. Between the Model and LGG groups, 17 differentially abundant genera were detected. Compared with the Model group, *Muribacter*, *Alistipes*, *Paraclostridium*, and *Tuzzerella* were significantly increased in the LGG group, whereas *Proteus* and *Rodentibacter* were significantly decreased (**Figure 2C**; **Supplementary Tables 1**, **2**).

ASV-level analysis further supported these findings (**Supplementary Figure 1**; **Supplementary Table 3**). In the comparison between the Control and Model groups, 69 significantly differential ASVs were identified, suggesting substantial ASV-level restructuring of the gut microbiota under rotavirus infection. Among these, several ASVs assigned to *Rodentibacter*, including ASV2, ASV4, and ASV6, showed increased relative abundance in the Model group, indicating that this taxon may contribute to disease-associated microbial dysbiosis. Further comparison between the Model and LGG groups identified 70 significantly differential ASVs. These changes involved multiple bacterial lineages, including *Rodentibacter*, *Proteus*, *Parabacteroides*, *Roseburia*, *Intestinimonas*, and *Enterococcus*, suggesting that LGG intervention not only altered the overall abundance of specific genera but may also reshape the gut microbial ecosystem at the higher-resolution ASV level.

### 3.3 LGG reshapes rotavirus-induced jejunoileal transcriptomic reprogramming

**Figure 3:**
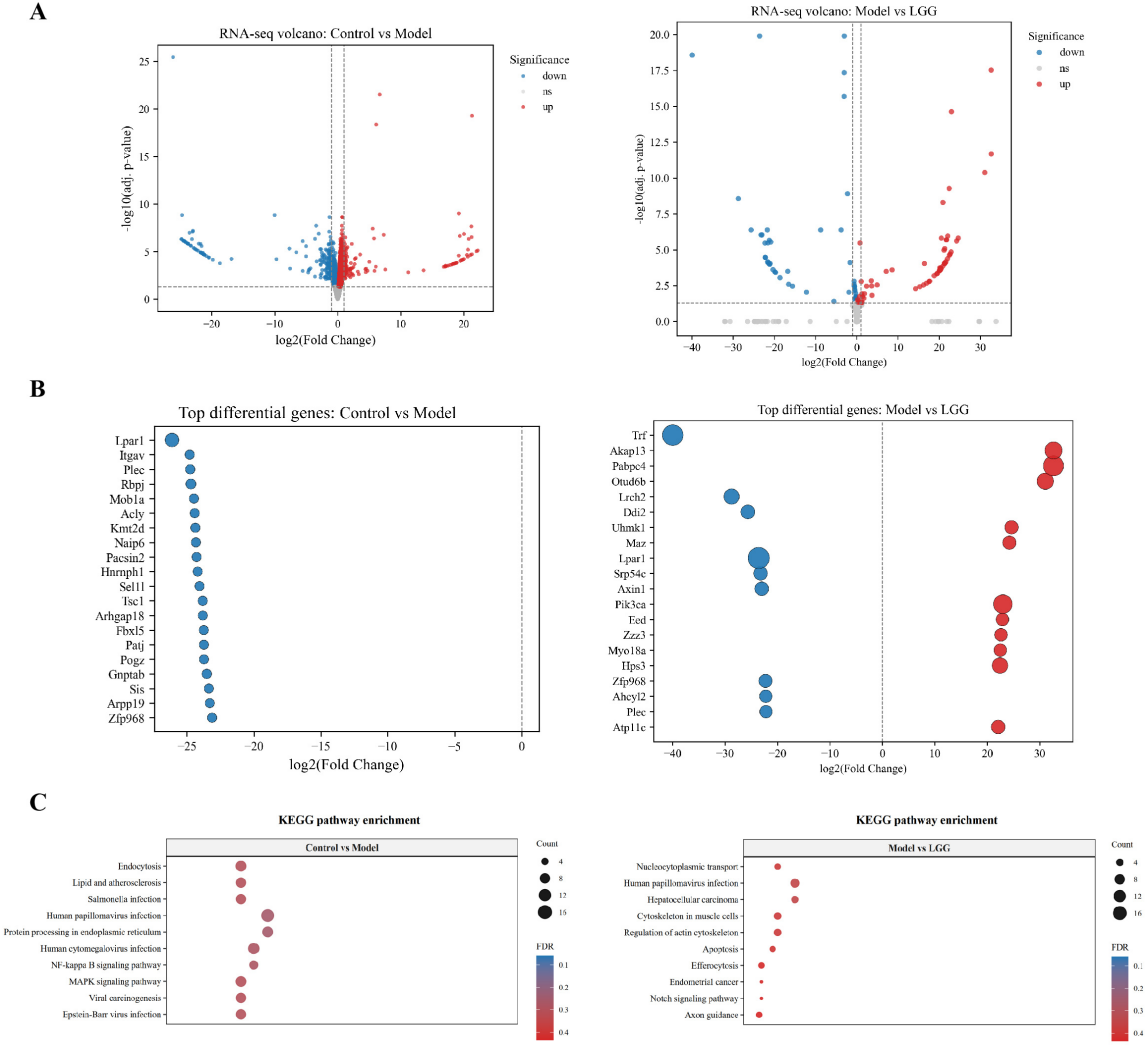
LGG reshapes rotavirus-induced jejunoileal transcriptomic reprogramming. **(A)** Volcano plot of differentially expressed genes. **(B)** Top differential genes between groups. **(C)** KEGG pathway enrichment.

RNA-seq analysis identified 323 and 91 stringently differentially expressed transcripts in the Control vs Model and Model vs LGG comparisons, respectively (**Figure 3A**). After rotavirus infection, genes such as *Lpar1*, *Inppl1*, *Rbpj*, *Srrm1*, and *Ikbkg* were markedly altered, whereas LGG intervention significantly affected the expression of *Lpar1*, *Trf*, *Rbpj*, *Axin1*, *Mysm1*, and other genes (**Figure 3B**). Further integrative analysis revealed 36 genes that were significantly different among the three groups (**Supplementary Table 4**).

Among these genes, the mucosal immunity-related genes *Ighv3–8* and *Ighv4–2* showed particularly prominent changes. Both genes were markedly upregulated after rotavirus infection and decreased toward levels observed in the normal control group following LGG intervention. As immunoglobulin heavy-chain variable region-related genes, they may reflect alterations in local B-cell activity and antibody receptor transcription. A second group of genes was associated with epithelial barrier function, cytoskeletal organization, and tissue repair, including *Lpar1*, *Amotl2*, *Arhgef28*, *Akap13*, *Mprip*, *Myo18a*, *Plec*, *Dysf*, and *Pacsin2*. Collectively, these genes were mainly related to cytoskeletal regulation, cell-cell junctions, membrane structural stability, and epithelial repair. A third group of genes was involved in oxidative stress, protein homeostasis, and cellular injury responses, including *Nfe2l1*, *Gstp2*, *Fbxl5*, *Aifm1*, *Dnajc7*, *Gnptab*, *Qars*, and *Otud6b*. Finally, *Rbpj* and *Axin1* were mainly associated with Notch/Wnt signaling and epithelial differentiation.

KEGG enrichment analysis showed that rotavirus infection induced pronounced infection-related responses, inflammatory activation, cellular stress, and abnormal protein processing in intestinal tissues (**Figure 3C**). Representative enriched pathways included endocytosis, protein processing in the endoplasmic reticulum, NF-kappa B signaling pathway, MAPK signaling pathway, and several virus infection-related pathways. After LGG intervention, differentially expressed genes in jejunoileal tissues were mainly enriched in pathways related to cellular structural remodeling, cell death and clearance, nucleocytoplasmic transport, and epithelial renewal. Representative pathways included regulation of actin cytoskeleton, cytoskeleton in muscle cells, apoptosis, efferocytosis, nucleocytoplasmic transport, and Notch signaling pathway.

### 3.4 LGG partially attenuates rotavirus-induced activation of mucosal B-cell/IgA related responses

**Figure 4:**
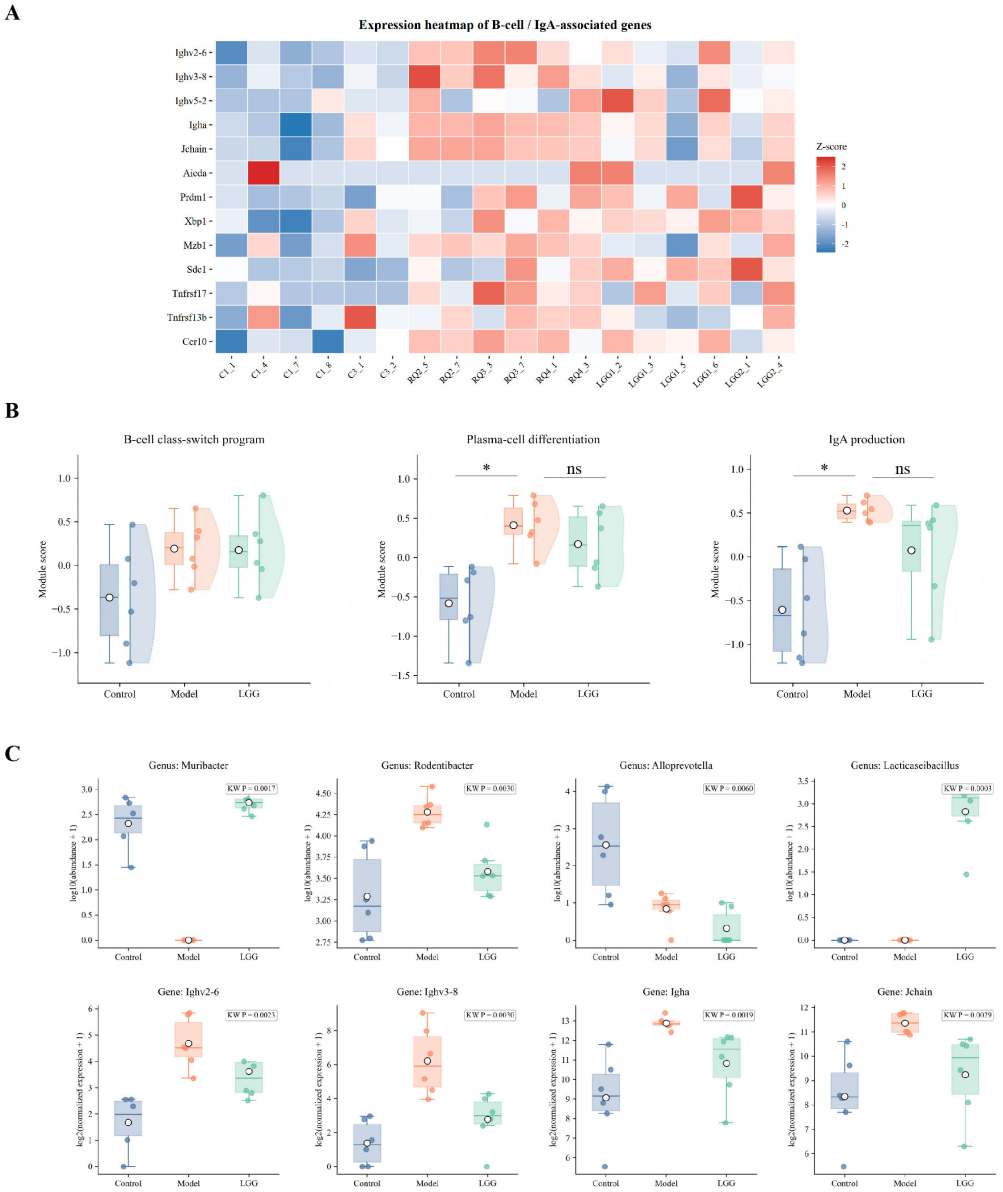
LGG partially attenuates rotavirus-associated activation of mucosal B-cell-IgA responses. **(A)** Expression heatmap of B-cell/IgA-associated genes. **(B)** Mucosal immunity-related module scores. **(C)** Coordinated alterations in gut bacterial genera and mucosal immunity-related genes. The symbol * means "P < 0.05", and ns indicates no significant difference.

Following rotavirus infection, genes associated with mucosal B-cell responses and IgA production were markedly activated (**Figure 4A**). Specifically, the expression levels of *Igha*, *Ighv10-1*, *Ighv2-6*, *Ighv2-9*, *Ighv3-5*, *Ighv3-8*, *Ighv4-2*, *Ighv5-9*, *Ighv9-3*, *Igkc*, *Igkv1-110*, *Igkv15-103*, *Igkv16-104*, *Igkv4-72*, and *Jchain* were significantly increased in the Model group. After LGG intervention, the expression levels of *Ighv3–8* and *Ighv4–2* were significantly decreased, whereas the remaining genes showed a downward trend without reaching statistical significance (**Supplementary Table 5**).

Based on these findings, we further calculated module scores for B-cell class switching, plasma-cell differentiation, and IgA production (**Figure 4B**). Compared with the Control group, the Model group showed increased scores across all three modules, with statistically significant differences observed for plasma-cell differentiation and IgA production. Compared with the Model group, the LGG group exhibited reduced scores for B-cell class switching, plasma-cell differentiation, and IgA production; however, none of these differences reached statistical significance. The sample-level heatmap was consistent with these results, showing an overall increase in the Model group relative to the Control group, whereas no clear separation was observed between the Model and LGG groups.

We further investigated the relationship between gut microbiota alterations and mucosal immune transcriptional responses (**Figure 4C**). After rotavirus infection, the relative abundances of *Muribacter* and *Alloprevotella* were markedly decreased, whereas *Rodentibacter* was increased. These microbial changes were accompanied by enhanced expression of B-cell/IgA-related genes, including *Ighv2-6*, *Ighv3-8*, *Igha*, and *Jchain*, suggesting that microbial dysbiosis coexisted with activation of mucosal humoral immunity during infection. Following LGG intervention, *Muribacter* was markedly restored, *Rodentibacter* was reduced, and *Lacticaseibacillus* was significantly enriched. Concurrently, most IgA/B-cell-related genes showed decreased expression or partial normalization relative to the Model group.

### 3.5 LGG modulates gut microbiota-host gene networks during rotavirus infection

**Figure 5:**
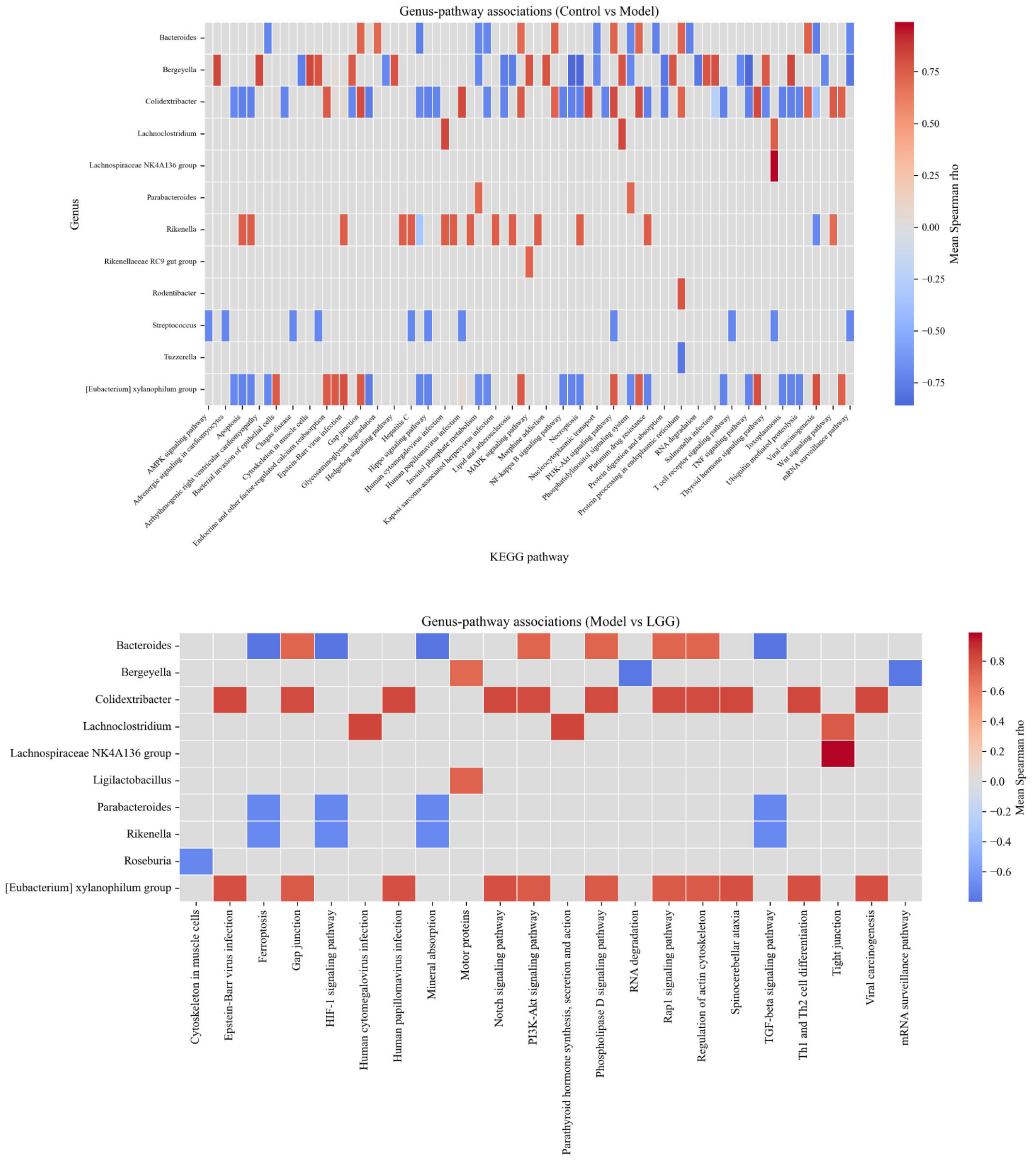
Genus pathway associations between groups.

To further elucidate the association between LGG-induced alterations in the gut microbiota and host intestinal transcriptional responses, we constructed an integrated Genus-Gene-Pathway network (**Figure 5**). A total of 645 significant genus-gene correlations were identified, including 265, 52, and 328 correlations in the Control vs Model, Model vs LGG, and LGG vs Control comparisons, respectively (**Supplementary Table 6**).

After rotavirus infection, the correlation network was mainly associated with inflammatory responses, immune regulation, cell death, and metabolism-related pathways. Representative association axes included the *Bacteroides*-*Inppl1*-B cell receptor/inositol phosphate/insulin signaling axis, the *Bergeyella*-*Dnm1l*-TNF/NOD-like receptor/necroptosis axis, and the *Colidextribacter*/*[Eubacterium] xylanophilum group*-*Birc3*/*Nfkb2*-NF-κB/TNF axis. Notably, *Bacteroides* was decreased in the Model group, whereas *Inppl1* was upregulated, with a negative correlation between them. Similarly, *Bergeyell*a was increased in the Model group, whereas *Dnm1l* was downregulated, and these two features were also negatively correlated.

After LGG intervention, microbial alterations were mainly associated with pathways related to intestinal epithelial barrier repair, cytoskeletal remodeling, Notch/immune differentiation, PI3K-Akt/Rap1 signaling, iron metabolism, and ferroptosis. Representative axes included the *Colidextribacter*-*Lpar1*-actin cytoskeleton/PI3K-Akt/Rap1/gap junction axis, the *Colidextribacter*/*[Eubacterium] xylanophilum group-Rbpj*-Notch/Th1-Th2 differentiation axis, the *Lachnospiraceae NK4A136 group*/*Lachnoclostridium*-*Amotl2*-tight junction axis, and the *Bacteroides*/*Parabacteroides*/*Rikenella*-*Trf*-ferroptosis/HIF-1/mineral absorption axis. Among these associations, *Colidextribacter* and *Lpar1* were synchronously increased after LGG intervention and showed a positive correlation. *Amotl2* was strongly positively correlated with the *Lachnospiraceae NK4A136 group* and was annotated to the tight junction pathway, indicating that this association may contribute to LGG-mediated improvement of intestinal mucosal barrier integrity.

## 4 Discussion

In this study, we established a neonatal mouse model of rotavirus infection and integrated 16S rRNA sequencing, jejunal-ileal transcriptomic profiling, and Genus-Gene-Pathway integrative analysis to investigate the associations between LGG-induced alterations in the gut microbiota and host intestinal transcriptional responses. The findings suggest that the protective effects of LGG against rotavirus enteritis may not depend solely on changes in a single bacterial genus or an individual immune parameter, but rather involve an integrated regulatory process characterized by gut microbiota remodeling, modulation of host transcriptional networks, epithelial barrier repair, and restoration of mucosal homeostasis.

Rotavirus infection significantly reduced the Shannon index and the number of observed ASVs, indicating that both the richness and evenness of the gut microbiota were disrupted under the disease condition, consistent with previous studies (Kim et al., 2020). Following LGG intervention, α-diversity showed an increasing trend, and the overall microbial community structure shifted toward that of the normal control state, although complete recovery was not achieved within the short observation period. These findings suggest that the effect of LGG may not primarily be manifested as a rapid restoration of overall microbial diversity, but rather as the remodeling of community composition and key ecological niches. Differential genus- and ASV-level analyses further supported this interpretation. The increased abundance of *Rodentibacter* and *Proteus* after infection may reflect intestinal barrier disruption, enhanced inflammatory responses, and expansion of facultative anaerobes, whereas the decreased abundance of *Alistipes*, *Paraclostridium*, and related taxa suggests perturbation of anaerobic metabolic niches. LGG intervention partially reversed these alterations and induced ASV-level rearrangements across multiple taxonomic lineages, including *Rodentibacter*, *Proteus*, *Parabacteroides*, *Roseburia*, *Intestinimonas*, and *Enterococcus*. These results suggest that LGG may promote the reconstruction of the intestinal microbial ecological network after infection through selective regulation at the strain or subpopulation level.

From the transcriptomic perspective, rotavirus infection induced marked transcriptional reprogramming in host intestinal tissues, involving infection-related responses, inflammatory activation, protein processing, endoplasmic reticulum stress, NF-κB/MAPK signaling, and virus infection-associated pathways. Activation of the NF-κB and MAPK pathways is generally associated with pro-inflammatory cytokine expression, epithelial injury responses, and immune-cell recruitment, whereas dysregulated protein processing in the endoplasmic reticulum may reflect the interplay among viral replication, protein synthesis stress, and epithelial cell damage. Following LGG intervention, the host response appeared to shift from “infection recognition and inflammatory activation” toward “tissue structural remodeling and post-injury repair.” In particular, genes such as *Lpar1*, *Amotl2*, *Arhgef28*, *Akap13*, *Mprip*, *Myo18a*, *Plec*, *Dysf*, and *Pacsin2* collectively pointed to the regulation of cytoskeletal organization, membrane structural stability, cell-cell junctions, and epithelial repair processes (Hultin et al., 2017; Lin et al., 2018; Postema et al., 2019; Krausova et al., 2021). Rotavirus infection can disrupt villous epithelial architecture and cell polarity, whereas reconstruction of the cytoskeleton and tight junctions is a critical step for restoring absorptive function and barrier integrity. Therefore, LGG-associated alterations in cytoskeletal and junction-related pathways may constitute an important molecular basis for its ability to alleviate diarrhea and intestinal pathological injury.

After rotavirus infection, *Igha*, *Jchain*, and multiple immunoglobulin variable region-related genes were significantly upregulated, accompanied by increased module scores for plasma-cell differentiation and IgA production. These findings indicate that rotavirus infection can induce a local mucosal humoral immune response, consistent with previous studies. Following LGG intervention, the module scores for B-cell class-switching, plasma-cell differentiation, and IgA production were reduced but did not reach statistical significance, and only *Ighv3–8* and *Ighv4–2* were significantly downregulated. These results suggest that the effect of LGG on the mucosal B-cell/IgA axis may be more closely related to modulation of infection-induced excessive or sustained inflammatory activation, rather than further enhancement of IgA-related gene expression. Previous clinical studies have shown that oral administration of LGG can shorten the disease duration in children with acute rotavirus diarrhea and increase rotavirus-specific IgA antibody-secreting cell responses during convalescence (Kaila et al., 1992). In addition, LGG has been reported to enhance the immunogenicity of oral rotavirus vaccines (Isolauri et al., 1995) and to modulate intestinal B-cell responses (Kandasamy et al., 2014), Th1-cell responses, and innate immune signaling in neonatal gnotobiotic pig models (Wang et al., 2016). However, the immunomodulatory effects observed across different studies are not entirely consistent. Subsequent studies have suggested that the enhancing effect of LGG on rotavirus vaccine-induced immune responses may be modest and context dependent (Wen et al., 2014; Lazarus et al., 2018). Therefore, in the present study, the decreased transcriptional activity of B-cell- and IgA-related programs after LGG intervention does not necessarily indicate impaired mucosal immune function. Rather, it may reflect a transition of the intestinal mucosal immune response from an inflammation-driven activated state toward immune homeostatic restoration.

The Genus-Gene-Pathway integrative analysis further revealed potential links between alterations in the gut microbiota and host transcriptional responses. Rotavirus-induced microbial dysbiosis may be closely associated with host inflammatory signaling, immune receptor signaling, cell death, and metabolic regulatory pathways. In particular, the increased abundance of *Bergeyella* together with the downregulation of *Dnm1l*, as well as their negative correlation, suggests a potential association between the expansion of specific bacterial taxa and mitochondrial dynamics, cell death, or inflammatory responses under infectious conditions. The associations of *Colidextribacter* and the *[Eubacterium] xylanophilum group* with *Birc3*, *Nfkb2*, and the NF-κB and TNF pathways further suggest that these dysbiotic taxa may participate in, or reflect, inflammatory remodeling of the intestinal mucosa after rotavirus infection. Following LGG intervention, the biological processes involved were mainly related to barrier repair, cytoskeletal remodeling, Notch signaling and immune differentiation, PI3K-Akt/Rap1 signaling, and iron metabolism/ferroptosis. *Lpar1* is involved in cellular migration, cytoskeletal organization, barrier repair, and epithelial regeneration. *Lpar1* and *Colidextribacter* were synchronously increased after LGG intervention and showed a positive correlation, suggesting that LGG-associated alterations in specific bacterial niches may be linked to host epithelial structural remodeling and restoration of cell - cell junctions. In addition, *Amotl2* was strongly positively correlated with the *Lachnospiraceae NK4A136 group* and was associated with the tight junction pathway, indicating a potential link between LGG-related microbial remodeling and the maintenance of tight junction integrity. Compared with separately describing changes in bacterial genus abundance or differential gene expression, integrated analysis of Genus–Gene–Pathway axes helps identify potential associations between LGG-induced alterations in gut microbial ecology and host functional recovery, thereby providing clues for understanding microbiota–host interactions.

## 5 Conclusion

This study suggests that LGG alleviates diarrhea symptoms and intestinal mucosal injury in neonatal mice with rotavirus infection, accompanied by remodeling of the gut microbiota composition and host transcriptional networks in the jejunoileum. Further multi-omics integration analysis showed coordinated changes between LGG-associated gut microbiota alterations and host transcriptional programs related to mucosal repair and immune homeostasis, suggesting that these microbiota–host associations may be involved in the restoration of intestinal mucosal homeostasis after rotavirus infection. These findings provide multi-omics evidence for understanding the potential microbiota–host interactions underlying LGG intervention in rotavirus enteritis and offer a reference for future mechanistic studies targeting gut microbial ecology and mucosal barrier repair.

### 5.1 Strengths and limitations

This study not only characterized alterations in gut microbial composition and host differentially expressed genes, but also constructed a Genus-Gene-Pathway integrative network, thereby linking changes in specific bacterial genera to host functional pathways and enhancing our understanding of microbiota-host interaction mechanisms. In addition, mucosal immunity-related modules were incorporated into the analysis. The finding that LGG intervention did not significantly enhance mucosal immunity-related transcriptional activity suggests that, in the context of acute viral enteritis, the immunomodulatory effects of LGG should not be simply interpreted as enhancement of mucosal immunity. Rather, they should be more cautiously understood as regulation of infection-associated immune activation and disruption of mucosal homeostasis.

However, several limitations should be acknowledged. First, an LGG-only treatment group was not included; therefore, we could not clearly distinguish whether the observed changes were attributable to the direct effects of LGG or were secondary to reduced disease severity, accelerated post-infection recovery, or enhanced viral clearance. This issue will be addressed in future studies. Second, the sample size of this study was relatively limited. Although histopathological changes showed a consistent trend, they did not reach statistical significance, and further validation in larger cohorts is warranted. Third, 16S rRNA sequencing mainly reflects changes in microbial composition and cannot adequately resolve strain-level differences or microbial functional outputs. Future studies integrating metagenomic sequencing, metatranscriptomics, or metabolomics are warranted to further clarify LGG-associated microbial functions. Forth, transcriptomic sequencing in this study was performed using bulk jejunal-ileal tissues, which does not allow discrimination of the contributions from different cell types, such as intestinal epithelial cells, immune cells, and stromal cells. Subsequent studies combining single-cell transcriptomics, spatial transcriptomics, or immunofluorescence localization may help validate the cellular sources of key genes and pathways. Fifth, the Genus-Gene-Pathway network was constructed based on correlation analysis and therefore cannot directly demonstrate that changes in bacterial genera cause alterations in host gene expression. Causal validation is still needed through experiments such as antibiotic-mediated microbiota depletion, fecal microbiota transplantation, supplementation with specific bacterial taxa, gene perturbation, or *in vitro* co-culture using intestinal epithelial organoids.

## Data Availability

The datasets presented in this study can be found in online repositories. The names of the repository/repositories and accession number(s) can be found in the article/**Supplementary Material**.

## Supplementary material

Supplementary Figure 1 ASV analysis. **(A)** Differentially abundant ASVs between groups; **(B)** Top ASVs between groups (log fold change).
