# Supplementary material for: Multi-omics analysis reveals gut microbiota-host transcriptomic remodeling associated with the protective effects of Lacticaseibacillus rhamnosus GG against rotavirus enteritis
Source: Front Cell Infect Microbiol. 2026 Aug 5;16:1901881. doi: 10.3389/fcimb.2026.1901881 (PMC13485510; doi:10.3389/fcimb.2026.1901881)
Supplement: Supplementary file 1 [file Table1.docx]

**Supplement**

**eFigure 1** ASV Analysis

**Table 1** Different Abundant Genera Between Groups

**Table 2** Top Different Genus Between Groups

**Table 3** ASV Analysis

**Table 4** Different Genes Between Groups

**Table 5** Genes Associated with Mucosal B-cell responses and IgA production

**Table 6** Genus-Gene-Pathway Analysis
